# Knowledge about cervical cancer in young Portuguese women: a cross-sectional study

**DOI:** 10.3389/fpubh.2024.1357606

**Published:** 2024-03-15

**Authors:** Inês Oliveira Rodrigues, Inês Duarte, Carolina Gomes Costa, Ana Luís Pimentel, Sílvia Chaves, Ana Patrícia Gomes, Lina Santos, Joana Isabel Santos, Ana Cristina Moreira, Andrea Lobão, Isabel Nazaré, Paulo Santos

**Affiliations:** ^1^USF Barão do Corvo, ULS Gaia, Vila Nova de Gaia, Portugal; ^2^Department of Community Medicine, Information and Health Decision Sciences, Faculty of Medicine, University of Porto, Porto, Portugal; ^3^Faculty of Medicine, Center for Health Technology and Services Research – CINTESIS@RISE, University of Porto, Porto, Portugal

**Keywords:** cervical cancer, human papillomavirus, cervical screening, attitude to health, literacy for health

## Abstract

**Introduction:**

Health literacy is crucial to adherence to medical interventions in therapeutics, prevention, and diagnosis. The basis for literacy is knowledge. To accomplish the goals for the elimination of cervical cancer, one of the most prevalent and preventable cancers, we must understand the determinants of non-adherence and address them specifically to ensure patients’ active participation.

**Aim:**

To determine women’s knowledge regarding the manifestations of cervical cancer and its prevention.

**Materials and methods:**

We conducted a cross-sectional study in an urban population from northern Portugal. Women aged 18 to 30 years were randomly assigned to answer the Cervical Cancer Awareness Measure questionnaire, including questions of knowledge about the causes and symptoms of cervical cancer, prospecting for individual and social-related determinants.

**Results:**

The total number of participants was 270, with a mean age of 24.7 years. Knowledge about symptoms scored 5.4 ± 2.6, with a maximum of 12 points, and knowledge about the causes scored 5.7 ± 1.9, with a maximum of 11 points. The correlation between both was 0.334. High education, high socio-economic status, self-perception of one’s capacity to recognize symptoms, and knowledge about the HPV vaccine were associated with better knowledge.

**Discussion:**

Portuguese women present low knowledge about cervical cancer, potentially affecting their health through exposure to risk situations and non-adherence to routine screening.

## 1 Introduction

Cervical cancer (CC) is one of the most common cancers in women (1). It has a slow and progressive development, with a prolonged pre-symptomatic phase. Human papillomavirus (HPV) infection is the leading risk factor for its genesis (2, 3). HPV DNA has been identified in 99.7% of invasive cancers. There is some worldwide variation in the prevalence of high-risk HPV genotypes, but more than 70% of squamous cell carcinomas and 90% of adenocarcinomas are related to types 16 and 18. The prevalence of HPV infection is significantly higher in women under 50 years old, where the rate of spontaneous resolution is also higher (4).

Globally, the incidence of neoplasms is increasing, including CC, which is the fourth most frequently diagnosed cancer and the fourth leading cause of cancer death in women (1, 5). As in most European countries, in Portugal, incidence and mortality have declined in recent years, primarily due to better genital hygiene, reduced parity, and a diminishing prevalence of sexually transmitted diseases (6). The incidence of CC is about 10.7 cases/100,000 women, similar to the European mean, with a mortality of 3.2/100,000, slightly below the 3.8 in European countries. The age group with the highest incidence of this cancer includes women between 40 and 49 years old (6).

The World Health Organization launched in 2020 a strategy for accelerating the elimination of cervical cancer based on the three pillars of vaccination, screening, and treatment. The aim is to vaccinate 90% of girls before 15 years old, to screen 70% of women aged 30–45 years old, to treat 90% of precancerous lesions, and to achieve 90% treatment and care for cervical cancer cases (7). From a health services point of view, it is crucial to create conditions for the success of these strategies, providing sound and equitable access to high-quality, innovative, and evidence-based medicine. On the other hand, we need to ensure patients’ adherence, under the perspective of one’s responsibility for their health, especially to preventive measures and screening. Health education is essential to increase the population’s knowledge about risk factors, clinical manifestations, and preventive measures related to the disease, including vaccination and screening. Lack of awareness is a significant constraint to screenings, potentially impacting its effectiveness (8). Knowledge about HPV and its role in cervical cancer is lower than expected, even in countries with established vaccination and screening programs (9). Unexpectedly, vaccinated girls and their mothers show low awareness of HPV and disease (10). Health literacy promotes the adoption of health-promoting behaviors and is an independent predictor of adherence to cancer screenings (11, 12).

Organized population-based screening for CC is a cost-effective secondary prevention measure (13). National Portuguese Health Authority recommends population-based screening in women between 25 and 60 years old every 5 years, using the detection of high-risk HPV nucleic acid in vaginal cytology. According to official data from 2019 to 2021, the population screening rates were 40–48%, and adherence among invited women was 76–94% (14).

We aim to characterize women’s knowledge regarding the manifestations of CC and its causes to intervene specifically in education for health programs directed to improve knowledge and skills for the reduction of the individual risk of cervical cancer.

## 2 Materials and methods

We conducted a cross-sectional study with an analytical component. The sample included women from 18 to 30 years old, corresponding to the ages of the most significant impact of preventive measures (6). Participants were enrolled at the Health Center of Barão do Corvo, a Primary Care Unit in northern Portugal, covering more than 13,000 people in the urban area of Vila Nova de Gaia. We excluded those who did not have sufficient knowledge of the Portuguese language to self-complete the questionnaire and who suffered from mental or other disorders that interfered with the autonomy for free, informed consent. We determined the minimum sample to be 270, based on the population size we sampled from (906), a 5% margin of error, and a 95% confidence interval (15).

We used a questionnaire based on the Cervical Cancer Awareness Measure (Cervical CAM). The University College London (UCL) Health Behavior Research Centre developed the Cervical CAM questionnaire in collaboration with the Department of Health Cancer Team and The Eve Appeal. It was a part of the Cervical Cancer Awareness and Symptoms Initiative (CCASI), based on the generic CAM developed by Cancer Research UK, University College London, King’s College London, and Oxford University in 2007–2008 (16), which showed good psychometric properties with high internal reliability (Cronbach’s *α* = 0.77) and test–retest reliability (*r* = 0.81). Questions were translated for Portuguese language, cultured adapted, and back translated by authors to assure correctness of concepts under evaluation. The variables included general demographic data, questions about knowledge and attitudes of CC preventive measures, adherence to anti-HPV immunization, and previous cervical-vaginal cytology. The questions about causes are answered on a 5-point Likert scale, categorized into a dichotomic answer by accepting the agree/completely agree as 1-true and disagree/completely disagree or not sure as 0-false. Socio-economic status was asked by a 10-point Likert scale from the lower self-estimation to the higher one, divided into low (1–3 points), medium (4–7 points), and high (8–10) social class. General health status perception and self-evaluation of health issues’ knowledge used a 5-point Likert scale, analyzed as continuous variables. We also included a variable about the previous education or experience in any health profession to adjust the model, as it is described to influence the knowledge about health issues. The self-administered questionnaire was tested in a pilot of a small group of women not included in the sampling.

The invitations were distributed by administrative staff among those eligible to participate in attending health center facilities for medical or nursing consultation on two previously determined random days per week for four consecutive weeks, from 6/02/2023 to 03/03/2023. A ballot box was available in the administrative sector of Barao do Corvo Health Centre to collect the questionnaires after answering, maintaining complete anonymity.

The procedures were approved by the Ethical Committee of North Health Administration (process number 64/2022).

The data was processed in a password-protected database using IBM SPSS^®^ version 27.

We used descriptive statistics: mean for continuous variables, standard deviation (SD), and proportions for categorical ones. The Kolmogorov–Smirnov test checked the normal distribution.

The quality standard was settled at 80% of correct answers (10 out of 12 for symptoms and 9 out of 11 for causes). The knowledge score on symptoms and causes of cervical cancer was calculated by simply adding the correct answers to each question, ranging between 1 and 12 for symptoms and 1 and 11 for causes of CC. For the multivariate analysis, the distribution was split by the median, considering the groups with the greatest and least knowledge, used as the dependent variable in the calculation of the logistic regression model, which was adjusted for the presence of training or experience in the health area.

## 3 Results

We included 270 women aged 18–30 (mean age = 24.7 ± 3.9 years). Women are mostly Portuguese (90.7%), single (72.6%), active workers (50.0%), with secondary school (50.0%), and from the socio-economic middle class (73.0%). A total of 38 (14.1%) presented with education or working in the health sector (Table 1).

**Table 1:** Socio-demographic characterization of the sample.

Characteristics		Total *n* = 270	(%)
Age (mean ± SD)		24.7 ± 3.9	
Nationality n (%)	Portuguese	245	90.7
	Other	13	5.1
	Unknown	12	4.4
Marital status n (%)	Single	196	72.6
	Other	70	25.9
	Unknown	4	1.5
Education n (%)	Basic: [0–9] years	19	7.0
	Secondary: [10–12] years	135	50.0
	Higher education	109	40.4
	Unknown	7	2.6
Working situation n (%)	Active workers	135	50.0
	Students	53	19.6
	Unemployed	62	23.0
	Others	2	0.7
	Unknown	18	6.7
Education or practice in the health sector n (%)		38	14.1
Self-reported socio-economic classificationn (%)	Low class	12	4.4
	Middle class	197	73.0
	High class	35	13.0
	Unknown	26	9.6
General health perception (1–5) (mean ± SD)		4.01 ± 0.77	

**Figure 1:**
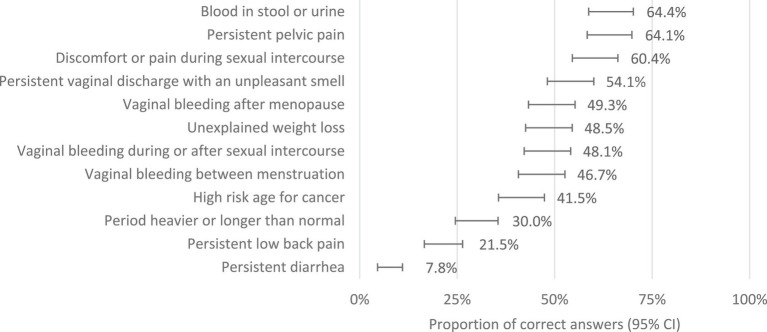
Identification of cervical cancer symptoms (proportion of women who correctly report the symptoms as being related to cervical cancer or not).

**Figure 2:**
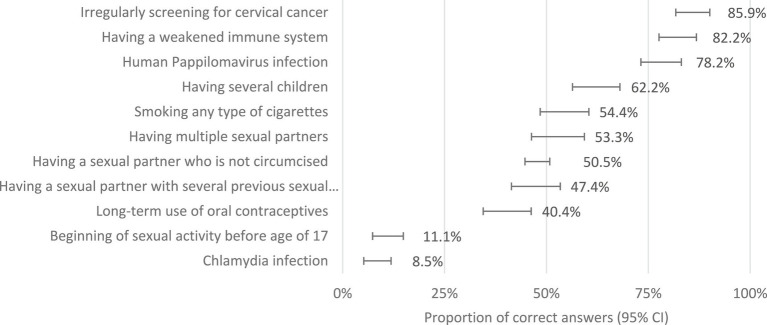
Identification of the causes of cervical cancer (proportion of women who correctly identify the causes as being or not related to cervical cancer).

The mean score of knowledge about symptoms of CC was 5.4 ± 2.6, in a maximum of 12 points. Only 10.0% of women (*n* = 27) answered 10 or more questions correctly. The presence of blood, pain, or vaginal discharge were the main symptoms pointed as potential suspicious of cervical cancer (Figure 1). However, just 49.3–64.4% identified it correctly. A total of 167 women (61.9%) presented four or more questions where they did not assume any answer, just saying “I do not know.” The mean score of the knowledge about the causes of CC was 5.7 ± 1.9 in a maximum of 11 points, with 18 women answering correctly above nine questions (6.7%). Human papillomavirus was identified as a cause of cervical cancer by 78.2% of women. They mostly tend to value the weakness of the immune system (82.2%) and the lack of screening (85.9%) as the leading causes (Figure 2). Regarding self-confidence in recognizing symptoms, the mean score was 2.54 (95% CI: 2.41–2.66) out of 5, where higher values correspond to higher self-confidence. In general, women with higher knowledge scores about causes tended to have higher knowledge scores about symptoms (Pearson correlation =0.33; 95% CI:0.22–0.44; *p* < 0.001).

The multivariate analysis allowed us to characterize the relation between knowledge about symptoms and causes of CC and several independent variables, using the median of each distribution to split women with high or low awareness. Education (*p* = 0.022), knowledge about the HPV vaccine (*p* = 0.001), self-perception of one’s capacity to recognize symptoms (*p* = 0.033), and understanding of the causes of CC (*p* = 0.007) are related to better knowledge about CC-related symptoms. On the other hand, knowing about the HPV vaccine (*p* = 0.009) and CC symptoms (*p* = 0.022) were associated with higher knowledge of the causes (Table 2).

**Table 2:** Multivariate analysis on conditions associated with knowledge of symptoms and causes.

Characteristics	Knowledge about the symptoms of cervical cancer			Knowledge about the causes of cervical cancer		
	OR	95%CI	*p*	OR	95%CI	*p*
Education or experience in healthcare	1.749	0.608–5.032	0.300	1.912	0.685–5.333	0.216
Single	1.237	0.551–2.777	0.607	0.568	0.253–1.268	0.167
Age	0.920	0.825–1.027	0.138	1.008	0.907–1.119	0.879
Portuguese	0.632	0.124–3.233	0.582	2.171	0.510–9.246	0.294
Education High Secondary Basic	<ref.> 0.528 0.110	– 0.262–1.062 0.02–0.617	0.022 0.073 0.012	<ref.> 0.553 0.455	0.151–2.028 0.116–1.790	0.522 0.372 0.260
Self-reported socioeconomic class Low Middle High	<ref.> 0.461 0.182	– 0.078–2.707 0.026–1.279	0.085 – 0.391 0.087	<ref.> 1.07 1.498	0.247–4.629 0.294–7.629	0.737 0.928 0.627
Labor condition Active worker Student Unemployed Other	<ref.> 1.144 0.686 0.975	– 0.41–3.193 0.296–1.589 0.247–3.852	0.761 – 0.797 0.379 0.971	<ref.> 0.507 0.553 0.783	0.197–1.300 0.250–1.222 0.201–3.046	0.392 0.157 0.143 0.724
Self-perception of own capacity to recognize symptoms	1.451	1.031–2.042	0.033	1.086	0.787–1.497	0.615
Knowledge about cervical cancer screening	1.528	0.725–3.222	0.265	1.192	0.590–2.408	0.624
Knowledge about HPV vaccine	4.000	1.718–9.313	0.001	2.673	1.282–5.568	0.009
Self-perception of literacy for health	1.256	0.788–2.002	0.338	0.750	0.486–1.156	0.192
Self-perception about health status	0.963	0.626–1.481	0.862	0.885	0.587–1.331	0.557
Knowledge of the causes of CC	1.316	1.077–1.609	0.007			
Knowledge of symptoms related to cervical cancer				1.162	1.022–1.322	0.022

## 4 Discussion

Our study shows that knowledge about the causes and symptoms of CC is low in most of our women since just 6.7 and 10.0% reached the desired standard of quality levels for causes and symptoms awareness. Symptom recognition in malignancies has been associated with a higher level of awareness and may lead to earlier seeking medical help (17). Knowing the causes and how to deal with them helps to make better choices and to change the adherence to prevention measures and screening (18). According to the International Agency for Research on Cancer, cervical cancer was the fourth most common cancer in females worldwide in 2020, accounting for 341,831 deaths. In Portugal, in the same year, it caused 379 deaths and was the 8th most common cancer (19). These are relevant numbers of one potentially preventable disease by behavioral changes and vaccination (5, 20).

In the literature, overall knowledge about symptoms of cervical cancer is low to moderate (21–23). The women in our study mentioned a median level of confidence in their ability to identify symptoms of cervical cancer (2.53 out of 5), with better perception associated with higher knowledge. All symptoms listed in the questions of the CAM may be, although not necessarily, warning signs for cervical cancer. Our participants showed doubts and a lack of knowledge about the symptoms, with many selecting the “I do not know” option. Persistent low back pain and diarrhea were, among the assessed symptoms, those less frequently reported by the respondents as symptoms related to cervical cancer. This may be explained by the fact that these symptoms are not recognized as usual gynecological symptoms. In contrast, intermenstrual bleeding, persistent vaginal discharge with an unpleasant smell, discomfort or pain during sex, vaginal bleeding after menopause, persistent pelvic pain, and vaginal bleeding during or after sex had a higher rate of recognition as potential symptoms of cervical cancer. Constitutional symptoms like unexplained weight loss were also identified by many women as possible symptoms, possibly because this symptomatology is commonly relatable to other malignancies. This pattern of answers was similar to those found in previous studies (21, 23).

Better knowledge about symptoms was associated with higher education, self-perception of one’s capacity to identify the symptoms and the knowledge about HPV vaccine, valuing the investment in education, especially taking advantage of the opportunities of contact with health services during the immunization procedures (20), and the known predisposition to vaccinate in better-informed women (24, 25).

Regarding the risk factors for cervical cancer, most participants identified HPV infection as a major cause. Nevertheless, they pointed to irregular screening and weakness of the immune system as the main causes. This pattern can lead to the idea of an individual’s lack of responsibility for the infection transmission mechanisms and, in some way, compromise its prevention. In the same sense, sexual behavior (starting to have sex at a young age, having many sexual partners, and having a sexual partner with several previous partners) presented lower scores (21, 26).

As with recognizing the symptoms, we found a significant association between knowing about the HPV vaccine and the score of symptoms, making it relevant to maximize the opportunities for intervention and health education, making it structural in the design of health care systems (27).

Educational interventions increase cervical cancer awareness, knowledge, and screening (5, 28, 29). Many studies showed the efficacy of interventions to increase the uptake of cervical cancer screening, including the utilization of reminders via phone call or SMS, the distribution of self-sampling HPV tests, and free, subsidized services. The combination with health education interventions improved the results (30). Health literacy is a predictor of participation in population-based screening programs for cervical cancer (12) and the key to implementing preventive medicine programs (11). e-Health solutions may be a good strategy for reaching a broad audience, but they must be framed by personalized contact with healthcare providers (31).

We identify some potential limitations in this study. Our participants were patients who visited the health center during the randomly selected days, which makes us cautious about the external validity to the population. Another question arises from our decision to include only Portuguese-speaking women because of the need to self-answer the questionnaire. In this region, as generally in the whole of the country, the great majority of foreign inhabitants come from Brazil and Portuguese-speaking African countries, which were included. Non-Portuguese-speaking women are very few, and their exclusion did not interfere significantly with the sampling validity. Nevertheless, it does not compromise our conclusions. On the contrary, despite the access to health services, we must work hard to improve their knowledge toward free and informed decision-making to enhance adherence to preventive measures, including cervical cancer screening. In Portugal, the recommendation for screening by pap smear starts at 25 years old. These participants were chosen specifically because they were near the age of starting or below, where the transmission of HPV is higher, and at least 10 years before the peak of cancer incidence, thus turning any intervention potentially more effective to change practices and to enhance adherence to screening procedures. These women are those who attend health center services, and we wonder how the rest of the population outside the health system is, making it essential to look forward to their ideas, misconceptions, and needs to improve the whole situation about cervical cancer in Portugal.

## 5 Conclusion

Portuguese women present low knowledge about cervical cancer, potentially affecting their health through exposition to risk situations and non-adherence to routine screening. Knowledge is central to health literacy, which is crucial for changing behaviors and adhering to preventive measures. We can hardly imagine an uninformed population being able to decide properly and freely, distinguishing evidence-based options from those more linked to marketing or unclear lobbying. Improving literacy should be a public health investment priority. Education for health is the beginning of changing this pathway, although we know that enhancing just information levels in the population is not enough to assure the attitudes and skills for better health. One step each time and the way will go through, with the support of healthcare providers, and mainly with the adherence of each one, which is really the main responsible for own health.

## Data availability statement

The raw data supporting the conclusions of this article will be made available by the authors, without undue reservation.

## Ethics statement

The studies involving humans were approved by Comissão de ética para a saúde da ARS-Norte, IP. The studies were conducted in accordance with the local legislation and institutional requirements. The participants provided their written informed consent to participate in this study.

## Author contributions

IR: Conceptualization, Formal analysis, Investigation, Writing – original draft, Writing – review & editing. ID: Conceptualization, Formal analysis, Investigation, Methodology, Writing – original draft, Writing – review & editing. CC: Conceptualization, Formal analysis, Investigation, Methodology, Writing – original draft, Writing – review & editing. AP: Conceptualization, Formal analysis, Investigation, Methodology, Writing – original draft, Writing – review & editing. SC: Conceptualization, Formal analysis, Investigation, Methodology, Writing – original draft, Writing – review & editing. AG: Conceptualization, Formal analysis, Investigation, Methodology, Writing – original draft, Writing – review & editing. LS: Conceptualization, Formal analysis, Investigation, Methodology, Writing – original draft, Writing – review & editing. JS: Conceptualization, Formal analysis, Investigation, Methodology, Project administration, Supervision, Writing – original draft, Writing – review & editing. AM: Supervision, Writing – original draft, Writing – review & editing, Conceptualization, Formal analysis, Investigation, Methodology, Project administration. AL: Conceptualization, Formal analysis, Investigation, Methodology, Writing – original draft, Writing – review & editing. IN: Conceptualization, Data curation, Formal analysis, Investigation, Methodology, Project administration, Supervision, Validation, Writing – original draft, Writing – review & editing. PS: Conceptualization, Data curation, Formal analysis, Funding acquisition, Investigation, Methodology, Project administration, Resources, Software, Supervision, Validation, Visualization, Writing – original draft, Writing – review & editing.
